# Supplementary material for: Impaired bone strength and bone microstructure in a novel early-onset osteoporotic rat model with a clinically relevant PLS3 mutation
Source: eLife. 2023 Apr 21;12:e80365. doi: 10.7554/eLife.80365 (PMC10159618; doi:10.7554/eLife.80365)
Supplement: Figure 1—source data 2. [file elife-80365-fig1-data2.zip › Figure 1source data 2 Western blotting analysis of PLS3 protein expression.pptx]

## Slide 1
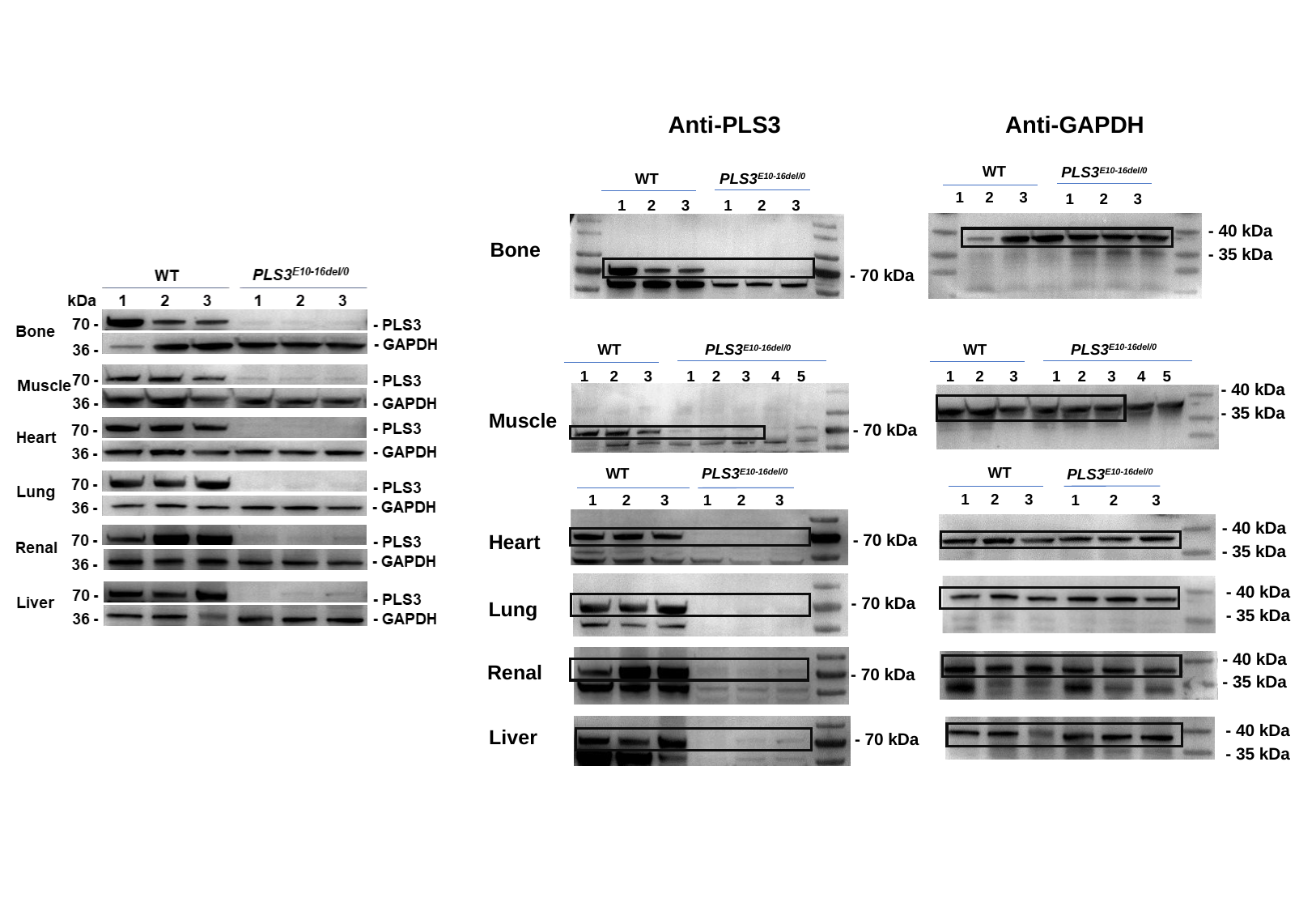

Anti-PLS3
Anti-GAPDH
WT
PLS3E10-16del/0
WT
PLS3E10-16del/0
 1 2 3
 1 2 3
 1 2 3
 1 2 3
- 40 kDa
Bone
- 35 kDa
- 70 kDa
WT
PLS3E10-16del/0
WT
PLS3E10-16del/0
 1 2 3
 1 2 3 4 5
 1 2 3
 1 2 3 4 5
- 40 kDa
- 35 kDa
Muscle
- 70 kDa
WT
WT
PLS3E10-16del/0
PLS3E10-16del/0
 1 2 3
 1 2 3
 1 2 3
 1 2 3
- 40 kDa
Heart
- 70 kDa
- 35 kDa
- 40 kDa
- 70 kDa
Lung
- 35 kDa
- 40 kDa
Renal
- 70 kDa
- 35 kDa
- 40 kDa
Liver
- 70 kDa
- 35 kDa
